# A Potent Lead Induces Apoptosis in Pancreatic Cancer Cells

**DOI:** 10.1371/journal.pone.0037841

**Published:** 2012-06-20

**Authors:** Zuojia Liu, Dan Li, Wenjing Zhao, Xiliang Zheng, Jin Wang, Erkang Wang

**Affiliations:** 1 State Key Laboratory of Electroanalytical Chemistry, Changchun Institute of Applied Chemistry, Chinese Academy of Sciences, Changchun, Jilin, China; 2 Department of Chemistry and Physics, State University of New York, Stony Brook, New York, United States of America; Wayne State University School of Medicine, United States of America

## Abstract

Pancreatic cancer is considered a lethal and treatment-refractory disease. To obtain a potent anticancer drug, the cytotoxic effect of 2-(benzo[d]oxazol-3(2H)-ylmethyl)- 5-((cyclohexylamino)methyl)benzene-1,4-diol, dihydrochloride (NSC48693) on human pancreatic cancer cells CFPAC-1, MiaPaCa-2, and BxPC-3 was assessed *in* *vitro*. The proliferation of CFPAC-1, MiaPaCa-2, and BxPC-3 is inhibited with IC_50_ value of 12.9±0.2, 20.6±0.3, and 6.2±0.6 µM at 48 h, respectively. This discovery is followed with additional analysis to demonstrate that NSC48693 inhibition is due to induction of apoptosis, including Annexin V staining, chromatins staining, and colony forming assays. It is further revealed that NSC48693 induces the release of cytochrome *c*, reduces mitochondrial membrane potential, generates reactive oxygen species, and activates caspase. These results collectively indicate that NSC48693 mainly induces apoptosis of CFPAC-1, MiaPaCa-2, and BxPC-3 cells by the mitochondrial-mediated apoptotic pathway. Excitingly, the study highlights an encouraging inhibition effect that human embryonic kidney (HEK-293) and liver (HL-7702) cells are more resistant to the antigrowth effect of NSC48693 compared to the three cancer cell lines. From this perspective, NSC48693 should help to open up a new opportunity for the treatment of patients with pancreatic cancer.

## Introduction

Pancreatic cancer is considered an aggressive cancer since it usually goes undetected until it reaches the late stage [1]. In 2010, pancreatic cancer was the 4^th^ most common cause of cancer-related death across the world [2]. Treatment of patients with pancreatic cancer mainly depends on surgery, radiation therapy, chemotherapy or combined therapeutic methods. It is well known that pancreatic cancer often has a poor prognosis since the 80-85% of patients present at a locally advanced or metastatic stage, precluding surgery, due to its high tendency for local invasion and distant metastases. Chemotherapy can be used to improve quality of life and gain a modest survival benefit of pancreatic cancer. Single-agent gemcitabine approved by FDA in 1998, represents currently the most effective chemotherapeutic drug that improves the quality of life and prolongs the life time of patients with advanced pancreatic cancer for 5-week. Due to the occurrence of chemoresistance to gemcitabine [3], gemcitabine-containing combinations, such as gemcitabine-erlotinib, -placebo and -oxaliplatin, were invented and exhibited synergistic effects and reduced resistance [4]. It is regretful that little convincing results are found on clinically relevant improvements in quality of life and survival. To address this issue, there appears to be an urgent need for the development of new anticancer drugs.

The understanding of the molecular mechanisms involved in the development of pancreatic cancer has provided many hopes for the discovery of new chemotherapeutical agents in pancreatic cancer therapy [5]. The inhibition of apoptosis plays a vital role in the deterioration process of pancreatic cancer like the other tumors [6]. Apoptosis, or programmed cell death, is a highly controlled physiological process and a core signaling pathway [7]. Thus, anticancer drugs as apoptotic inducer has been proposed and widely accepted in the therapy of cancers [8]. Some works have revealed that apoptotic-mediating therapy is a promising horizon for the therapy in cancers with little toxicity to surrounding normal cells due to their physiologically controlled survival pathway [9], [10]. The apoptosis-inducing therefore continues to represent an important direction for the development of new drugs for the treatment in patients with pancreatic cancer.

**Figure 1:**
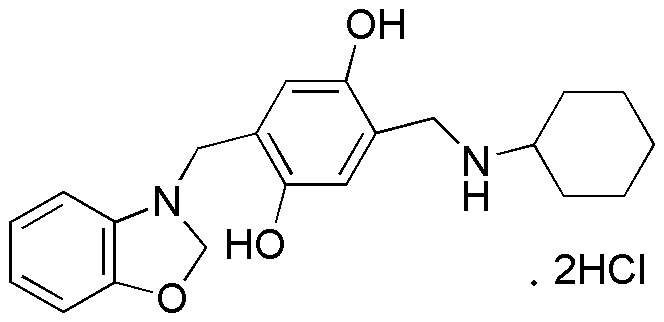
The structure of NSC48693.

In the present study, a potent lead termed as 2-(benzo[d]oxazol-3(2H)- ylmethyl)- 5-((cyclohexylamino)methyl)benzene-1,4-diol, dihydrochloride (NSC48693,Fig. 1) inhibits the proliferation of pancreatic cancer cells CFPAC-1, MiaPaCa-2, and BxPC-3 by inducing apoptosis in the mitochondrial-mediated pathway *in* *vitro*. Excitingly, the cytotoxic effect of NSC48693 on cancer cells is more pronounced than normal human embryonic kidney (HEK-293) and liver (HL-7702) cells. These results support a role for NSC48693 as a potent apoptotic inducer of pancreatic cancer.

## Materials and Methods

### Materials

All chemicals including Ac-DEVD-fmk (caspase-3 inhibitor) and Ac-LEHD-fmk (caspase-9 inhibitor) were purchased from Sigma (St Louis, MO), unless otherwise indicated. DMEM,IMDM and fetal bovine serum (FBS) were purchased from Gibico (Grand island, NY). The primary antibodies against caspase-3, caspase-8, caspase-9, Bcl-2, Bax, cytochrome *c* and peroxidase-conjugated goat antimouse or antirabbit secondary antibody as well as isotype mouse IgG1, goat anti-mouse IgG1 FITC were purchased from BD Bioscience (San Jose, CA). NSC48693 was kindly supplied from NCI/DTP Open Chemical Repository (http://dtp.cancer.gov). NSC48693 was dissolved in DMSO to make stock solution (10 mg/mL) and diluted to various concentrations with double distilled water containing 10% DMSO.

### Background of NSC48693

The extensive studies on pancreatic cancer have identified that Ras signaling is involved in the regulation of apoptosis. The development of drugs targeting Ras and inducing apoptosis is being pursued intensively in drug discovery. The active GTP-bound Ras is in equilibrium with three distinct states, one of which is the open non-signaling conformation that is a transient intermediate during GTP hydrolysis [11]. This implies that the Ras intermediate is a convergent point for survival signaling in pancreatic cancer. At present, therefore, the open conformation appears to be the most promising target for drug design. The structure of GppNHp-bound RasG60A-GTP (PDB ID: 1XCM [11]) was used in the docking calculations. All docking calculations were performed by using the Autodock package [12]. The database of National Cancer Institute (NCI) diversity set was used for the virtual screening. We then ranked these small molecules according to the predicted affinity and specificity defined [13]. 2-(benzo[d]oxazol-3(2H)-ylmethyl)-5-((cyclohexyl- amino)-methyl)benzene-1,4-diol, dihydrochloride (NSC48693) picked out from NCI database demonstrated growth inhibition effect on leukemia cell lines CCRF-CEM and MOLT-4 as shown in NCI Cancer Screen Current Data (http://dtp.nci.nih.gov). Little efforts focus on the effect of NSC48693 on pancreatic cancer, thus it offers a selected choice of high quality inducer of apoptosis.

### Cell Culture

The human pancreatic cancer cell lines CFPAC-1, MiaPaCa-2, and BxPC-3 were obtained from American Type Culture Collection (ATCC, Rockville, MD) and cultured in DMEM and IMDM medium supplemented with 10% FBS and antibiotics (100 units/mL penicillin and 100 µg/mL streptomycin sulfate), respectively. The human embryonic kidney 293 (HEK-293) and liver (HL-7702) cells were obtained from Chinese Academy of Science Type Culture Collection (Shanghai, China) and incubated in DMEM medium supplemented with 10% FBS. The cells were detached from the monolayer using 0.25% trypsin and 0.53 mM EDTA for 5 min at 37°C when the cells were grown to near confluence.

### Hoechst 33342 Staining

The three human pancreatic cancer cells (1×10^5^ cells/plate) were respectively seeded onto 6-well glass-bottomed plate and allowed to attach overnight. The cells cultured in 135 µL DMEM medium were treated by using either 15 µL 250 µg/mL NSC48693 (final concentration 25.0 µg/mL) as experimental groups or 15 µL double distilled water containing 10% DMSO as control groups, and then cultured for 48 h at 37°C and 5% CO_2_ conditions. Thereafter, the cells were fixed in MeOH–HOAc (3∶1, v/v) for 10 min at 4°C and then stained using Hoechst 33342 kit (KeyGEN Biotech, Nanjing, China). The stained cells were analyzed by confocal-laser scanning microscope (TCS SP2, Heidelberg, Germany).

### Cytotoxicity Assays

The cell viability of three pancreatic cancer cells and two human normal cells (1×10^4^ cells/well in 96-well plate) after being treated by double distilled water containing 10% DMSO as control groups or various concentrations of NSC48693 as experimental groups was assessed by thiazolyl blue tetrazolium bromide (MTT) assay. The cells were treated for 48 h and then the optical density (OD) at 490 nm was read with a 96-well multiscanner autoreader (Biotech Instruments, New York). MTT does not interfere with NSC48693 and causes a positive response.

### Soft Agar Assays

Soft agar assays were performed essentially as previously described [14]. The single cell suspensions of pancreatic cancer cells containing 1×10^4^ cells in 0.3% agar were placed in 3.5 cm dishes on top of a gelled layer of 1% agar in medium (DMEM or IMDM with 10% FBS) and cultured with double distilled water containing 10% DMSO as control groups or various concentrations of NSC48693 as experimental groups at 37°C. Colonies were fixed with 2.5% glutaraldehyde and counted scoring only the colonies that were larger than 10 cells in diameter under Olympus X71 inverted phase microscope (Dr. Schumann Optik OHG, Hessen, Germany). Percentage of colony forming efficiency was calculated as the number of colonies/100 seeded cells.

### Apoptosis Assays

The apoptosis of three pancreatic cancer cells (1×10^6^ cells/well in 24-well plate) after being treated by double distilled water containing 10% DMSO as control groups or various concentrations of NSC48693 as experimental groups was measured by Annexin V-FITC/propidium iodide (PI) apoptosis detection kit (KeyGEN Biotech, Nanjing, China). The quantification of PI and FITC signals was performed using fluorescence activated cell sorter FACSAria (BD Bioscience, San Jose, CA) and percentage of stained cells in each quadrant was quantified using Diva 6.0 software (BD Bioscience, San Jose, CA). In total, 10,000 events were analyzed in each sample.

### Caspase Activity Assay

Cells were cultured in 6-well plate (5% CO_2_, 37°C) at seeding density of 2×10^6^ cells/well. Plates were preincubated overnight before the solutions of drug were added to each well. After a period of exposure (24 or 48 h) with various concentrations NSC48693, cells harvested by trypsinization with trypsin/EDTA solution were washed by PBS solution and the activity of caspase-3, caspase-8, and caspase-9 was determined by caspase activity assay kit (KeyGEN Biotech, Nanjing, China), respectively. DEVDase, IETDase, and LEHDase activity was measured by measuring proteolytic cleavage of chromogenic substrates Ac-DEVD-pNA, Ac-IETD-pNA, and Ac-LEHDpNA, which were used as the substrates for caspase-3, caspase-8, and caspase-9-like proteases, respectively [15]. The absorbance of enzymatically-released pNA was measured at 490 nm on a multiscanner autoreader.

**Figure 2:**
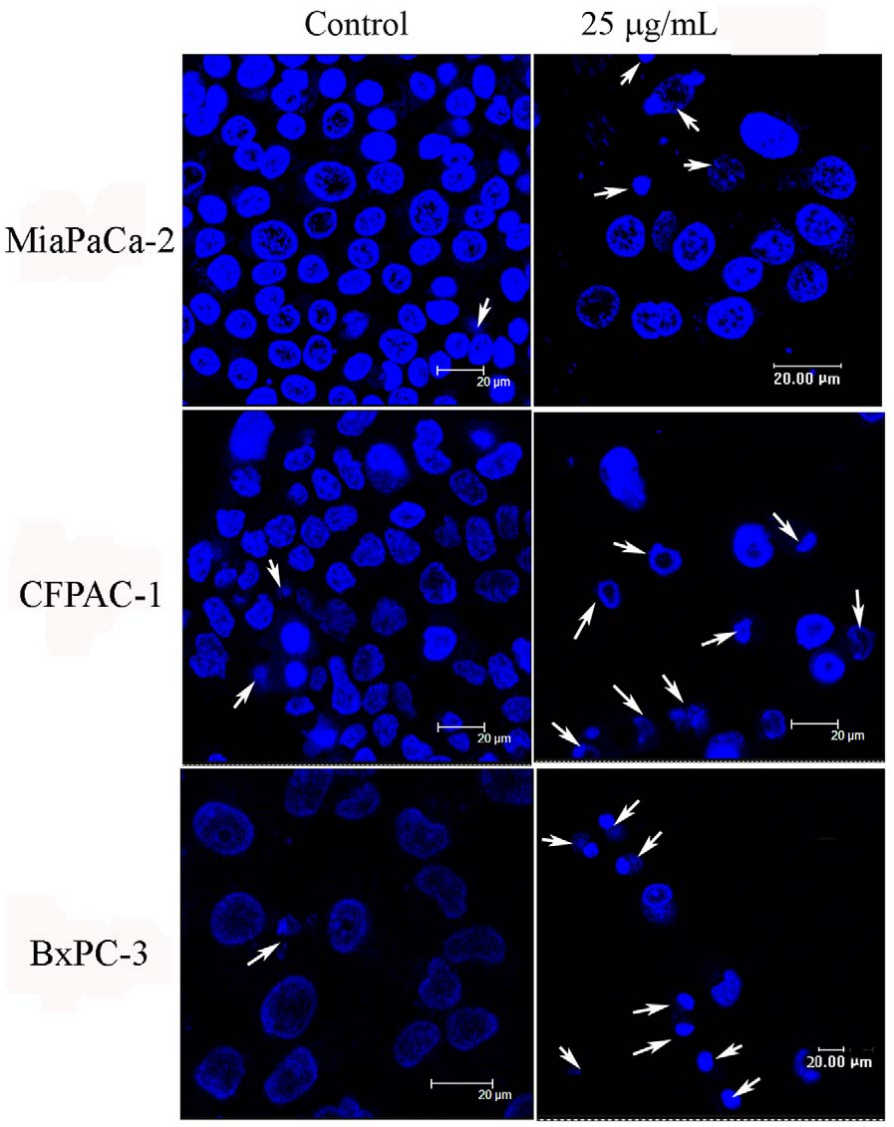
NSC48693 induces morphological alterations in CFPAC-1, MiaPaCa-2, and BxPC-3 cells. After 24 h adherence, the cells (1×10^5^ cells/plate) were treated with 25.0 µg/mL of NSC48693 for 48 h. Then, cells were examined using confocal laser scanning microscopy.

### Analysis of Cytochrome *c* Release

Cells were cultured in 24-well plate (5% CO_2_, 37°C) at seeding density of 5×10^4^ cells/well. Plates were preincubated overnight before the solutions of drug were added to each well. After a period of exposure (6 or 12 h) with various concentrations NSC48693, cells were harvested by trypsinization with trypsin/EDTA solution and washed with Hanks balanced salt solution supplemented with 2% bovine serum albumin and 0.2% azide. Thereafter, cells were fixed and permeabilized using Cytofix/Cytoperm kit (BD Bioscience, San Jose, CA). Unspecific binding of anti-cytochrome *c* antibody was blocked with mouse IgG1. Anti-cytochrome *c* (7H8.2C12) was added for additional 30 min at 4°C. Then, the cells were incubated with goat anti-mouse IgG1 FITC (1∶20) for 30 min at 4°C. After washing with Perm/Wash solution, cells were immediately analyzed by flow cytometry (FCM). Totally, 10,000 events per sample were acquired and analyzed.

### Measurement of the Mitochondria Membrane Potential (ΔΨm)

Changes in ΔΨm were measured by Rhodamine-123 (Rho-123) dye [16]. Cells were cultured in 24-well plate (5% CO2, 37°C) at seeding density of 5×10^4^ cells/well. Plates were preincubated overnight before the solutions of drug were added to each well. After a period of exposure (48 h) with various concentrations NSC48693, cells harvested by trypsinization with trypsin/EDTA solution were washed by PBS solution and incubated with Rho-123 (10 µg/mL). Thereafter, Rho-123 fluorescence was measured by FCM. The percentage of Rho-123- cells represents the effective collapsed ΔΨm; the reduction of Rho-123 indicates the loss of ΔΨm in cells [16]. In total, 10,000 events were analyzed in each sample.

### Detection of Reactive Oxygen Species (ROS)

Flow cytometric detection of ROS was performed as described previously [17]. Briefly, cells were cultured in 24-well plate (5% CO_2_, 37°C) at seeding density of 5×10^4^ cells/well. Plates were preincubated overnight before the solutions of drug were added to each well. After a period of exposure (2 h) with various concentrations NSC48693, cells were harvested by trypsinization with trypsin/EDTA solution and subsequently ROS fluorescence intensity was determined by FCM. ROS levels were expressed as percentage, which was calculated by Diva 6.0 software. In total, 10,000 events were analyzed in each sample.

**Figure 3:**
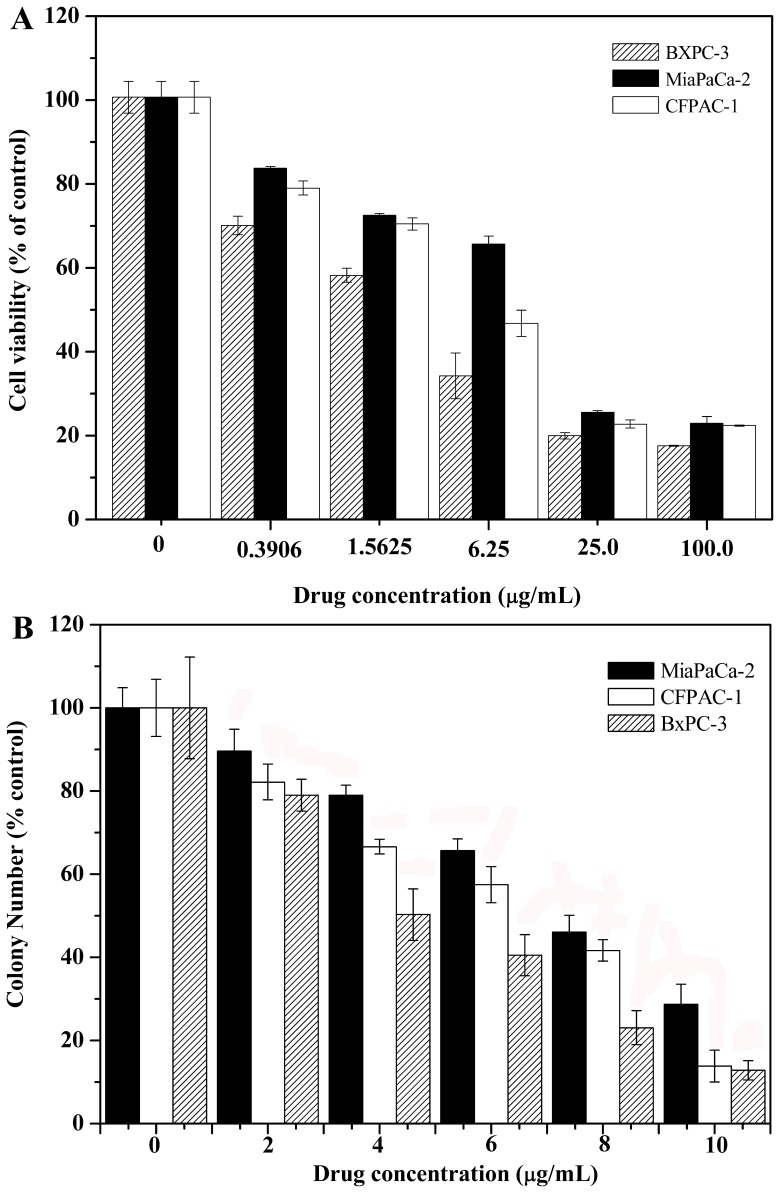
NSC48693 inhibits the proliferation of CFPAC-1, MiaPaCa-2, and BxPC-3 cells. After 24 h adherence, the cells (1×10^4^ cells/well in 96-well plate or 1 ×10^4^ cells in 3.5 cm dish) were treated with various concentrations of NSC48693 as indicated for 48 h. Then, the growth inhibition was assessed by MTT method (A) and anchorage independent growth assays (B), respectively. Each value represents mean ± SD in three independent experiments.

### Western Blotting

The cells (2×10^6^ cells) were seed to 10 cm plate and cultured in the incubator overnight. Then the cells were exposed with 1 or 2× IC_50_ concentration of NSC48693 and 10% DMSO (control) for 24 h. Whole-cell proteins and mitochondrial fractions were isolated by using the protein extraction kit (KeyGEN Biotech, Nanjing, China). The protein concentrations were determined using BCA protein assay kit (KeyGEN Biotech, Nanjing, China). Equal amounts of proteins were fractionated using 12% SDS-PAGE and then transferred to the polyvinylidene difluoride (PVDF) membranes. The membranes were incubated with primary antibodies against caspase-3, caspase-8, caspase-9, cytochrome *c*, Bcl-2, and Bax, after being blocked with 3% BSA in TBS. The membranes were incubated with peroxidase-conjugated goat antimouse or antirabbit secondary antibody. Actin was used for the control loading. In the inhibition assay for caspase-3 and caspase-9, the cells were pretreated by 20 µM Ac-DEVD-fmk or Ac -LEHD-fmk for 2 h after adherence, and the medium was changed by the culture containing 2× IC_50_ concentration of NSC48693 or 10% DMSO.

### Statistical Analysis

All data were expressed as means ± SD of triplicate experiments, and reproducibility was confirmed in at least three separate experiments. Statistical analysis was done using SPSS 11.5 statistical software.

**Figure 4:**
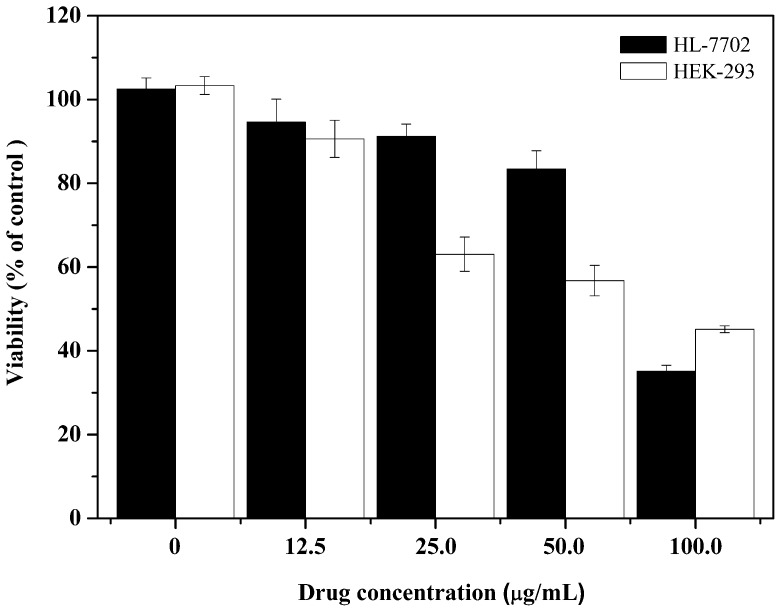
The cytotoxic effect of NSC48693 on normal HEK-293 and HL-7702 cells. After 24 h adherence, the cells (1×10^4^ cells/well in 96-well plate) were treated with different concentrations of NSC48693 as indicated for 48 h. Then, the growth inhibition assays were assessed by MTT method. Each value represents mean ± SD in three independent experiments.

**Figure 5:**
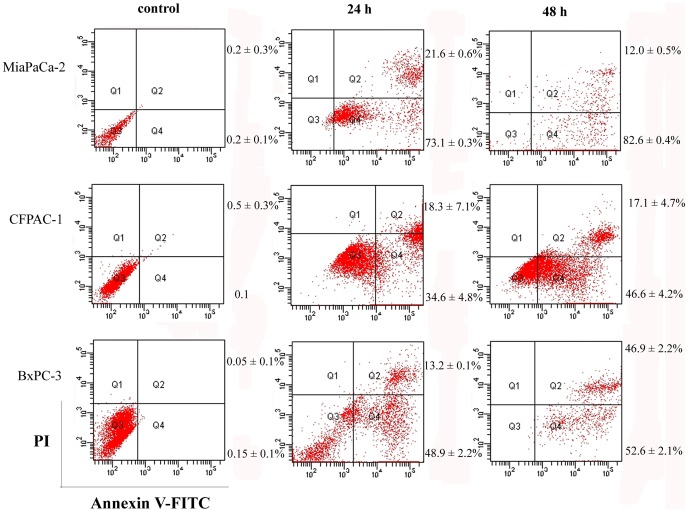
NSC48693 induces apoptosis in CFPAC-1, MiaPaCa-2, and BxPC-3 cells. Extent of apoptosis induced in cancer cells (1×10^6^ cells/well in 24-well plate) treated by 25.0 µg/mL of NSC48693 in MiaPaCa-2, 12.5 µg/mL of NSC48693 in CFPAC-1, and 6.25 µg/mL of NSC48693 in BxPC-3 for 24 or 48 h was measured by FCM, respectively. Representative results from three independent experiments are shown; and each value represents mean ± SD in three independent experiments.

## Results

### NSC48693 Changes Cell Morphology

Cellular morphology can be used as a parameter for measuring the effect of a compound on cell toxicity. Since the nuclear condensation occurs in this stage of apoptosis, the apoptotic morphology of the nucleus will be evident upon staining [18]. The nuclear staining with Hoechst 33342 shows that the cells in control groups are proportional and well distributed (Fig. 2). As compared with the normal nuclear morphology, typical morphological changes, including cytoskeletal collapse, the formation of apoptotic bodies and nuclear fragmentation, are observed in the treated cells as shown with arrows in Fig. 2. These characteristics consistently occurred during this form of cell death, which were all the common characters of cell death [19].

**Figure 6:**
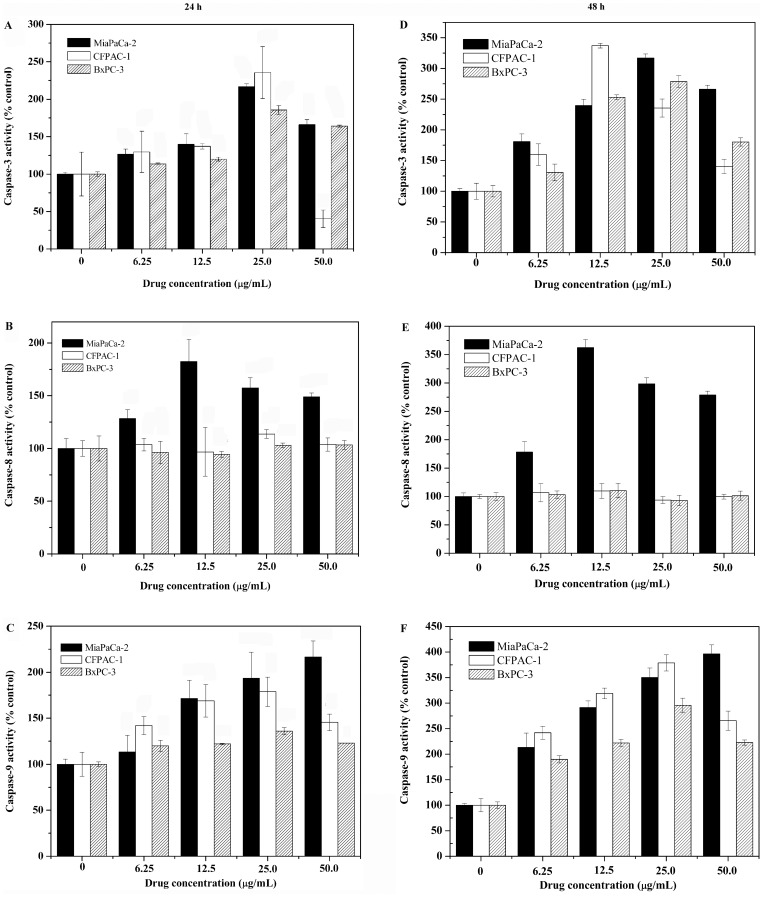
Effect of NSC48693 on the activation of caspase-3, caspase-8, and caspase-9 in CFPAC-1, MiaPaCa-2, and BxPC-3 cells. Cells (2×10^6^ cells/well in 6-well plate) were treated with NSC48693 at the indicated concentrations for 24 (A, B, C) or 48 h (D, E, F) then examined. Each value represents mean ± SD in three independent experiments.

**Figure 7:**
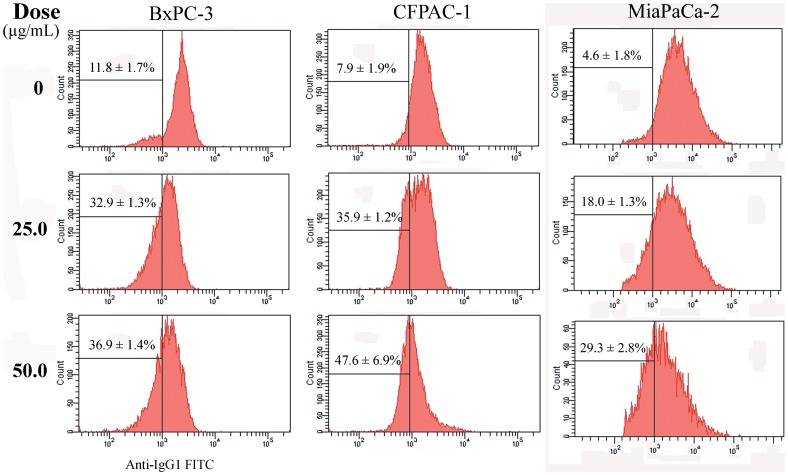
Analysis of mitochondrial cytochrome *c* release. The cells (5×10^4^ cells/well in 24-well plate) were treated at the indicated doses for 6 (CFPAC-1 and BxPC-3) or 12 h (MiaPaCa-2). Intracellular cytochrome *c* was detected by antibody 7H8.2C12 in fixed and permeabilzed cells. Percentages represent the proportion of cells with lowered cytochrome *c* signal. Representative results from three independent experiments are shown; and each value represents mean ± SD in three independent experiments.

### NSC48693 Inhibits Cell Proliferation

In addition to detailed comparative analysis of the morphological changes, the cytotoxicity of NSC48693 to cells was detected through the loss of cell viability using MTT assay [20]. As shown in Fig. 3A, the viability of cancer cells is significantly decreased by NSC48693 with the exposure dose increased. NSC48693 inhibits cancer cells proliferation with IC_50_ value of 12.9±0.2 µM for CFPAC-1, 20.6±0.3 µM for MiaPaCa-2, and 6.2±0.6 µM for BxPC-3 at 48 h, respectively. This was further supported by the ability of NSC48693 to block the anchorage-independent growth of cancer cells in soft agar. When compared with control groups, cancer cells form smaller and fewer colonies when grown in the presence of NSC48693. As indicated in Fig. 3B, this response occurs in a dose-dependent manner. Combined with the results, we conclude that NSC48693 decreases the proliferation of cancer cells in a dose-dependent manner.

### Cytotoxic Effects of NSC48693 on HEK-293 and HL-7702

As showed in Fig. 4, the cell viability of HEK-293 and HL-7702 is 63.4±4.1% and 91.2±2.9% at a dose of 25.0 µg/mL of NSC48693, respectively. NSC48693 inhibits HEK-293 and HL-7702 cells proliferation with an IC_50_ value 144.5±8.8 µM and 198.6±11.3 µM at 48 h, respectively. Obviously, the cytotoxic effects of NSC48693 on normal human cells are less sensitive than cancer cells CFPAC-1, MiaPaCa-2, and BxPC-3 as compared to Fig. 3.

**Figure 8:**
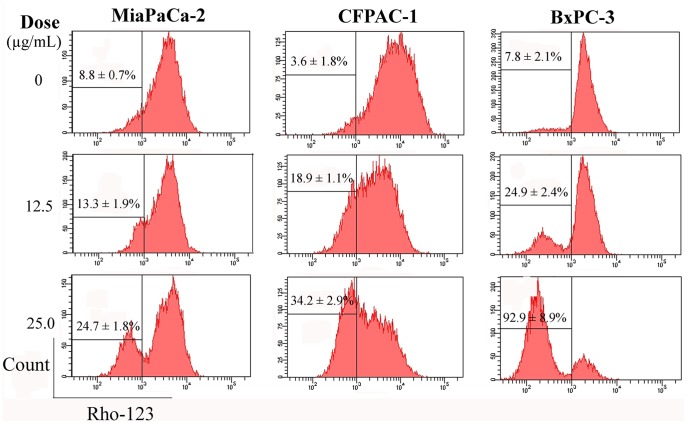
Analysis of the loss of ΔΨm. The cells (5×10^4^ cells/well in 24-well plate) were treated at the indicated doses for 48 h. Then the experiments were subject to FCM. Representative results from three independent experiments are shown; and each value represents mean ± SD in three independent experiments.

### NSC48693 Induces Apoptosis

To rule out the possibility that inhibition of cell growth was due to cytotoxic effects, apoptosis assay was performed. Cells positive for Annexin V-FITC and negative for PI are in early stage of apoptosis as shown in Q4 quadrant, while cells positive for both Annexin V-FITC and PI are in the late stage of apoptosis or necrosis as shown in Q2 quadrant [21]. Thus, the degree of apoptosis correlated with the amount of positive Annexin V-FITC cells. And the intact membrane integrity assessed by negative PI staining suggests that apoptosis but not necrosis is the character of apoptosis. As shown in Fig. 5, there is little binding of Annexin V-FITC to the control cells, while the binding after treatment with NSC48693 is increased as shown in Q4 quadrant. As for MiaPaCa-2 cells treated at the concentration of 25.0 µg/mL of NSC48693, the binding is increased from 73.1±1.0% to 82.7±2.8% at 24 h and 48 h. The binding is increased from 37.1±2.0% to 50.5±3.1% at 24 h and 48 h, after CFPAC-1 cells are treated by 12.5 µg/mL of NSC48693. While the binding is increased from 46.4±2.4% to 55.0±1.1% at 24 h and 48 h, after BxPC-3 cells are treated by 6.25 µg/mL of NSC48693. Combined with these observations, it is clear that the effect of NSC48693 on cancer cells is primarily mediated by inducing apoptotic cell death.

### Activation of Caspase

As major mediators of apoptosis, the activation of caspase depends on proteolytic cleavage of the procaspase. To better our understanding whether caspase was involved in the NSC48693-induced apoptosis in cancer cells, we examined enzyme activities of caspases with a spectrofluorometric assay using their corresponding substrates [22]. As shown in Fig. 6A, C, D, F, the activity of caspase-3 and caspase-9 is increased in a dose-and time-dependent manner compared with untreated cells in three cancer cells; the activity of caspase-8 linked to the death receptors fails to be increased in CFPAC-1 and BxPC-3 cells (Fig. 6B, E). However, the activity of caspase-8 in MiaPaCa-2 cells is increased in a dose-and time-dependent manner compared with untreated cells. These findings imply that NSC48693 activates the intrinsic mitochondria-mediated apoptotic pathway leading to caspase activation to induce apoptosis in CFPAC-1 and BxPC-3 cells. For MiaPaCa-2 cells, NSC48693 activates both the mitochondrial and membrane death receptor apoptotic pathway.

**Figure 9:**
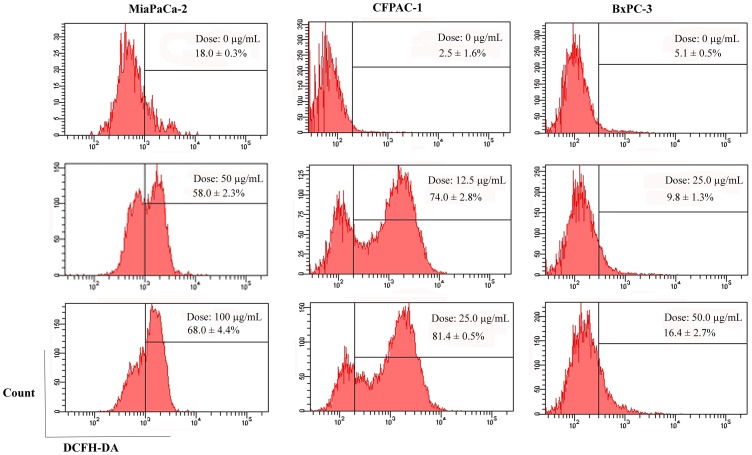
Analysis of the generation of ROS. The cells (5×10^4^ cells/well in 24-well plate) were treated at the indicated doses for 2 h. Then the experiments were subject to FCM. Representative results from three independent experiments are shown; and each value represents mean ± SD in three independent experiments.

**Figure 10:**
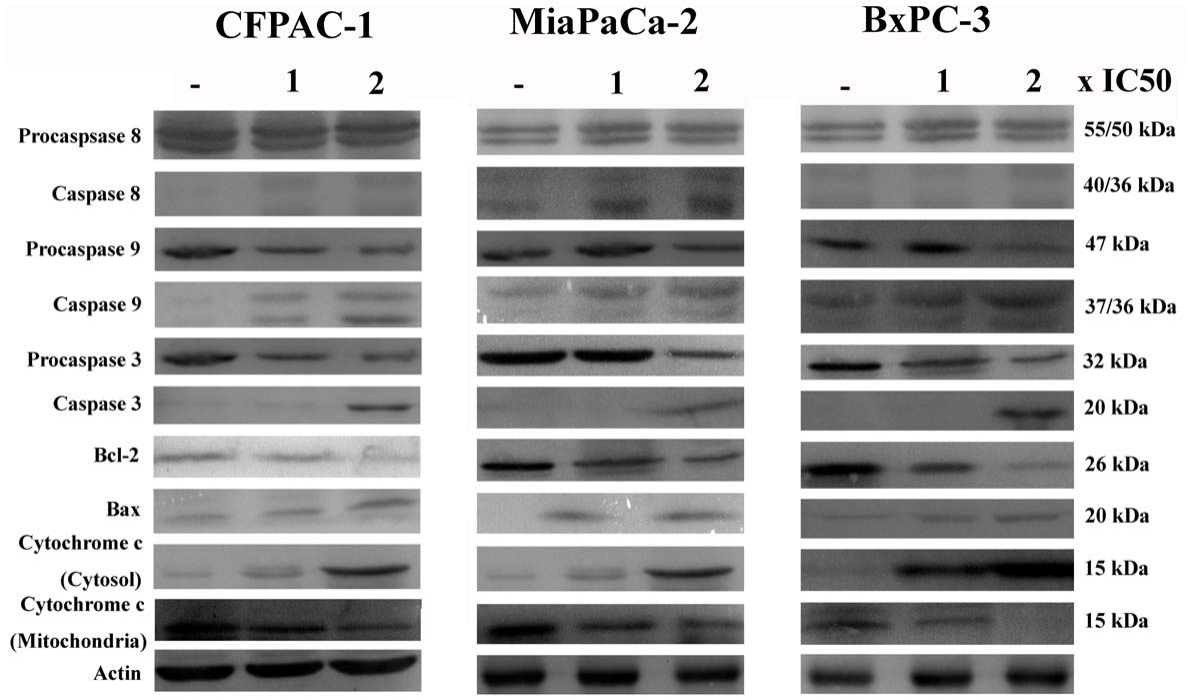
Immunoblots of whole-cell protein extracts after exposure to vehicle or to 1 or 2×IC_50_ compound concentration for 24 h in CFPAC-1, MiaPaCa-2, and BxPC-3 cells. Equal amounts of cellular proteins were fractionated on 12% SDS-PAGE gels and transferred to PVDF membranes. Actin was used to control loading.

### Release of cytochrome *c*

The release of cytochrome *c* is a crucial step controlling the apoptosome pathway. Clone 7H8.2C12 has been chosen to specially detect intracellular cytochrome *c*. Therefore, the reduction in cytochrome *c* signal detected by antibody 7H8.2C12 reflects mitochondrial cytochrome *c* release and early-onset of apoptosis [23]. As shown in Fig. 7, BxPC-3 cells in control groups have low level of cytochrome *c* (11.8±1.7%), whereas NSC48693 treatment enhances mitochondrial cytochrome *c* release from 32.9±1.3% to 36.9±1.4% at the concentrations of 25.0 and 50.0 µg/mL for 6 h. CFPAC-1 cells untreated by NSC48693 have normal mitochondrial cytochrome *c* level (7.9±1.9%), while the mitochondrial cytochrome *c* release levels are increased from 35.9±1.2% to 47.6±0.6.9% at the concentrations of 25.0 and 50.0 µg/mL for 6 h. Also, MiaPaCa-2 cells treated by NSC48693 have an enhanced mitochondrial cytochrome *c* release from 4.6±1.8% in the control group to 19.0±1.3% and 29.3±2.8% at the concentration of 25.0 and 50.0 µg/mL for 12 h, respectively.

### Loss of ΔΨm

ΔΨm can be produced when protons were pumped from the mitochondrial matrix to the inter-mitochondrial space [24]. And the dissipation of ΔΨm has been linked to some apoptotic pathways, including mitochondrial apoptotic pathway [25]. Absolutely most untreated cells have intact plasma membrane and normal ΔΨm, however, the loss of ΔΨm exhibits a dose-dependent increase manner after cells were treated with NSC48693 at 12.5 and 25.0 µg/mL for 48 h (Fig. 8). After MiaPaCa-2 cells being treated with NSC48693, the percentage of ΔΨm collapse reaches 13.3±1.9% and 24.7±1.8%, respectively. And the percentage of ΔΨm collapse of CFPAC-1 cells reaches 18.9±1.1% and 34.2±2.9%, respectively. While the percentage of ΔΨm collapse in BxPC-3 cells is obvious and reaches 24.9±2.4% and 92.9±8.9%, respectively. The experiments provide a sufficiently accurate basis for the conclusion that cancer cells treated by NSC48693 lose ΔΨm.

**Figure 11:**
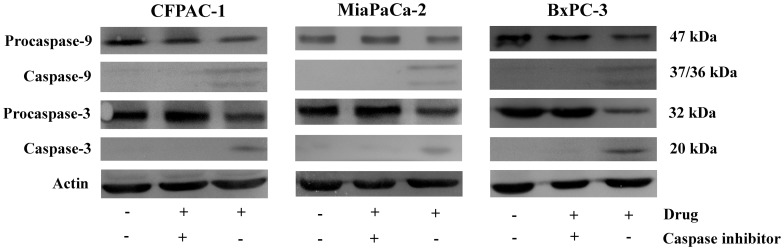
Effect of caspase-3 and caspase-9 inhibitor on NSC48693-induced apoptosis for 24 h in CFPAC-1, MiaPaCa-2, and BxPC-3 cells. Equal amounts of cellular proteins were fractionated on 12% SDS-PAGE gels and transferred to PVDF membranes. Actin was used to control loading.

### Generation of ROS

The generation of ROS is directly related to mitochondria [16]. As demonstrated in Fig. 9, flow cytometric analysis reveals that MiaPaCa-2 cells untreated have low level of endogenous ROS (18.0±0.3%), whereas NSC48693 treatment enhances significantly intracellular ROS levels from 58.0±2.3% to 68.0±4.4% at the concentrations of 50.0 and 100.0 µg/mL for 2 h. CFPAC-1 cells untreated by NSC48693 have normal endogenous ROS level (2.5±1.6%), while the ROS levels are increased from 74.0±2.8% to 81.4±0.5% at the concentrations of 12.5 and 25.0 µg/mL for 2 h. Also, the ROS levels are increased from 9.8±1.3% to 16.4±2.7% after BxPC-3 cells are treated by NSC48693 at the concentrations of 25.0 and 50.0 µg/mL for 2 h.

### Effect of NSC48693 on Apoptosis-Related Proteins

The effect of NSC48693 on apoptosis-related proteins was confirmed by western blotting as shown in Fig. 10. NSC48693 treatment increased the cleavage of caspase-9 and caspase-3 in a dose-dependent manner. The expression of cleaved-caspase-8 was almost undetectable in CFPAC-1 and BxPC-3 cells. However, the cleavage of caspase-8 was obvious in MiaPaCa-2 cells, which was agreement with the caspase activity as shown in Fig. 6. Simultaneously, the Bcl-2 protein expression was down regulated and Bax was up regulated. The cytochrome *c* in cytosol was enhanced concomitant with the related attenuation of cytochrome *c* in mitochondria. In addition, the specific inhibitors of caspase-9 and caspase-3 almost completely abolished the NSC48693-induced cleavage of caspase-9 and caspase-3, respectively (Fig. 11).

## Discussion

Although the uncovered chemotherapeutic drugs have yet to achieve any real progress in clinical therapy so far, the chemotherapy is still a backbone in the treatment of advanced pancreatic cancer at present [25]. Thus, novel chemotherapeutic drugs with different modes of action are always in need for the treatment of pancreatic cancer patients. The mechanism of cell death induced by chemotherapeutic drugs is thought to be an endogenous apoptotic mechanism. Thus the induction of apoptosis in cancer cells has been recognized as an innovative drug discovery strategy for cancer therapy [26]–[28].

Potential apoptotic inducers should kill cancer cells instead of causing excessive toxicity to normal ones. In the present study, we focused on evaluating the effect of NSC48693 on the death by inducing apoptosis of human pancreatic cancer cells CFPAC-1, MiaPaCa-2, and BxPC-3 *in* *vitro*. It appears that cancerous and normal cells react differently to NSC48693 exposure in MTT assays. The results of cell viability indicate that the viability of cancer cells is significantly inhibited by NSC48693 with IC50 of 12.9±0.2 µM for CFPAC-1, 20.6±0.3 µM for MiaPaCa-2, and 6.2±0.6 µM for BxPC-3, respectively. However, the sensitivity of normal cells to NSC48693 is considerably different. In contrast, no obvious cytotoxicity is detected in normal cells with IC_50_ 144.5±8.8 µM for HEK-293 and 198.6±11.3 µM for HL-7702, respectively. These results provide direct evidence that normal cells HEK-293 and HL-7702 are more resistant to the anti-growth effects of NSC48693 compared to the cancer cells CFPAC-1, MiaPaCa-2, and BxPC-3. Based on the growth inhibitory effect caused by NSC48693 on cancer cells as shown in Fig. 3, the cell apoptosis in response to NSC48693 was investigated. Fig. 5 suggests that NSC48693 triggers the apoptotic cell death of pancreatic cancer cells, which implies that NSC48693 has higher cytotoxicity via inducing marked apoptotic cell death in cancer cells. Moreover, different cell types vary profoundly in their susceptibility to apoptosis induction, which leas to distinct cellular thresholds existing for apoptosis induction [29]. The critical issue is surrounding normal tissues or cells may pause and repair the damage while tumor cells die through apoptosis. NSC48693 appears to be less toxic to normal cell lines, which may attribute to this possibility that normal cells have some protective mechanisms against NSC48693 [30]. During the normal cellular reduction, hydrogen peroxide generates and hydroxyl radical occurs. These can be lost through uncoupling or driven off, leading to oxidative damage. The normal cells have protective mechanisms to detoxify excessive ROS, leading to the reduction of the toxic effect of NSC48693 on HEK-293 and HL-7702.

Apoptosis can be triggered by several stimuli, and mitochondria are considered to play a central role in both caspase-dependent and caspase-independent apoptosis [31]. Mitochondria initiate two distinct apoptosis pathways, namely the intrinsic mitochondrial pathway and extrinsic membrane death receptor pathway [32]. For the major of chemotherapeutic drugs, apoptosis is initiated by the intrinsic mitochondrial pathway [27], [28]. Thus, understanding the regulation of the intrinsic mitochondrial apoptotic pathway is important for developing new inducer of apoptosis and verifying the induction of apoptosis. Many pro-death stimuli converge on the mitochondrial pathway during apoptosis, which leads to permeabilization of the outer mitochondrial membrane and the release of proteins resided in the mitochondrial inter membrane space. Cytochrome *c*, a kind of soluble apoptosis signaling molecules, localized in the mitochondrial intermembrane space is released into the cytosol upon apoptosis induction as shown in Fig. 7 and Fig. 10. The cytochrome *c* release from the cells is an apoptosis-specific process after induction of apoptosis, but not during necrosis [33].

Loss of ΔΨm was observed under NSC48693 treatment as a dose-dependent manner, suggesting that mitochondria are affected particularly early during the apoptotic process. In the process of apoptosis, mitochondria are a source of ROS generated by the reduced ΔΨm [34]. And the enhancement of ROS production has long been related to the apoptotic response induced by anti-cancer agents [35]. The relationship between ROS and the potency inducing apoptosis in NSC48693-induced cancer cells as shown in Fig. 9 indicates that NSC48693 elevates intracellular ROS levels.

To dissect the apoptotic cascade initiated by NSC48693-induced dissipation of ΔΨm, the proteolytic activity of caspase-3 and caspase-9 that is a marker for the mitochondrial pathway of apoptosis was quantified. The dissipation of ΔΨm activated downstream effectors caspase-3 and caspase-9, with an increase in a dose- and time-dependent manner (Fig. 6A, C, D, F). The little up-regulation of caspase-8 activity other than MiaPaCa-2 as shown in Fig. 6B, E ruled out the possible participation of death receptors [36]. Rather, these observations confirmed that the process of dissipation of ΔΨm was essential for the initiation and activation of downstream events with caspase-3 and caspase-9 culminated in apoptotic cell death [37]. These results implied that ROS release in the presence of caspase activation was sufficient for effective apoptosis of cancer cells.

Together with the data detailed in this study, several lines of evidence suggest that NSC48693 works as an agent inducing apoptosis in cancer cells by mainly activating the mitochondrial-mediated apoptotic pathway. With more follow-up experiments, therefore, studies on the molecular mechanisms of NSC48693 action should help to shed light on the treatment of pancreatic cancer.
